# Fetuin-A Induces Cytokine Expression and Suppresses Adiponectin Production

**DOI:** 10.1371/journal.pone.0001765

**Published:** 2008-03-12

**Authors:** Anita M. Hennige, Harald Staiger, Corinna Wicke, Fausto Machicao, Andreas Fritsche, Hans-Ulrich Häring, Norbert Stefan

**Affiliations:** 1 Department of Internal Medicine, Division of Endocrinology, Nephrology, Vascular Disease and Clinical Chemistry, University of Tübingen, Tübingen, Germany; 2 Department of General, Visceral and Transplantation Surgery, University of Tübingen, Tübingen, Germany; University of Parma, Italy

## Abstract

**Background:**

The secreted liver protein fetuin-A (AHSG) is up-regulated in hepatic steatosis and the metabolic syndrome. These states are strongly associated with low-grade inflammation and hypoadiponectinemia. We, therefore, hypothesized that fetuin-A may play a role in the regulation of cytokine expression, the modulation of adipose tissue expression and plasma concentration of the insulin-sensitizing and atheroprotective adipokine adiponectin.

**Methodology and Principal Findings:**

Human monocytic THP1 cells and human *in vitro* differenttiated adipocytes as well as C57BL/6 mice were treated with fetuin-A. mRNA expression of the genes encoding inflammatory cytokines and the adipokine adiponectin (ADIPOQ) was assessed by real-time RT-PCR. In 122 subjects, plasma levels of fetuin-A, adiponectin and, in a subgroup, the multimeric forms of adiponectin were determined. Fetuin-A treatment induced *TNF* and *IL1B* mRNA expression in THP1 cells (p<0.05). Treatment of mice with fetuin-A, analogously, resulted in a marked increase in adipose tissue *Tnf* mRNA as well as *Il6* expression (27- and 174-fold, respectively). These effects were accompanied by a decrease in adipose tissue *Adipoq* mRNA expression and lower circulating adiponectin levels (p<0.05, both). Furthermore, fetuin-A repressed *ADIPOQ* mRNA expression of human *in vitro* differentiated adipocytes (p<0.02) and induced inflammatory cytokine expression. In humans in plasma, fetuin-A correlated positively with high-sensitivity C-reactive protein, a marker of subclinical inflammation (r = 0.26, p = 0.01), and negatively with total- (r = −0.28, p = 0.02) and, particularly, high molecular weight adiponectin (r = −0.36, p = 0.01).

**Conclusions and Significance:**

We provide novel evidence that the secreted liver protein fetuin-A induces low-grade inflammation and represses adiponectin production in animals and in humans. These data suggest an important role of fatty liver in the pathophysiology of insulin resistance and atherosclerosis.

## Introduction

Fetuin-A (former name for the human protein: α_2_-Heremans-Schmid glycoprotein, AHSG) is an abundant serum protein [1] that is exclusively produced by the liver, tongue, and placenta [2]. In several studies, fetuin-A was shown to act as a natural inhibitor of the insulin receptor tyrosine kinase in liver and skeletal muscle [3]–[7]. In addition, mice deficient for the gene encoding fetuin-A displayed improved insulin sensitivity and were resistant to weight gain upon a high-fat diet [8]. Besides these well-documented effects of fetuin-A on the insulin receptor of muscle and liver, another mechanism of this protein may include effects on adipose tissue to induce whole-body insulin resistance. Recently, polymorphisms in the gene encoding human fetuin-A were found to be not only associated with type 2 diabetes [9], but also to affect insulin action in adipocytes [10]. Furthermore, fetuin-A was shown to exert direct pro-adipogenic properties [11], however, the underlying mechanisms are unknown.

The genes encoding human fetuin-A (*AHSG*) and human adiponectin (*ADIPOQ*), which is almost exclusively secreted from adipose tissue and represents an important determinant of whole-body insulin sensitivity [12]–[16] and cardiovascular disease [17]–[20], are located next to each other on chromosome 3q27. This chromosomal region was previously mapped as a type 2 diabetes and metabolic syndrome susceptibility locus [21], [22], and also shows close linkage to variation in plasma adiponectin levels [23]. However, not all of the variability in plasma adiponectin levels can be explained by genetic variation of the *ADIPOQ* gene [23]. Therefore, other genes under this linkage peak may encode proteins regulating adiponectin production with *AHSG* representing a major candidate.

Recently, we and others have shown that human plasma fetuin-A levels are correlated with fatty liver, impaired glucose tolerance, and insulin resistance [24], [25]. Moreover, a recent study provided evidence that human plasma fetuin-A levels are strongly associated with the metabolic syndrome and an atherogenic lipid profile [26]. Since these states are characterized by subclinical inflammation and hypoadiponectinemia [27], and based on the chromosomal localization of *AHSG*, we investigated whether fetuin-A regulates cytokine expression, modulates adipose tissue expression and the plasma concentration of the insulin-sensitizing and atheroprotective adipokine adiponectin.

## Material and Methods

### Cell culture

Human THP1 monocytes were obtained from American Type Culture Collection (Manassas, VA, USA) and cultured in IMDM supplemented with 10% (v/v) FCS, 100 IU/ml penicillin, 0.1 mg/ml streptomycin, and 2 mmol/l glutamine. Cells were left untreated or stimulated for 8 and 24 h with 600 µg/ml human fetuin-A (purity ≥95%, Sigma-Aldrich, Steinheim, Germany), a high physiological dose, or human serum albumin for control (ZLB Behring, Marburg, Germany), respectively.

Human preadipocytes were isolated from abdominal subcutaneous fat biopsies as previously described [28]. The donors underwent abdominal surgery for clinical purposes and gave informed written consent to the study. Isolated preadipocytes were expanded in α-MEM/Ham's nutrient mixture F12 (1:1) containing 20% (v/v) FCS, 1% (v/v) chicken embryo extract (Sera Laboratories International, Horsted Keynes, UK), 100 IU/ml penicillin, 0.1 mg/ml streptomycin, 0.5 µg/ml fungizone, and 2 mmol/l glutamine. First-pass cells were used for the experiments. At confluence, adipose conversion was induced by shifting the cells into DMEM/Ham's nutrient mixture F12 (1:1), 5% (v/v) FCS, 17 µmol/l pantothenate, 1 µmol/l biotin, 2 µg/ml apo-transferrin, 1 µmol/l insulin, 100 IU/ml penicillin, 0.1 mg/ml streptomycin, 0.5 µg/ml fungizone, and 2 mmol/l glutamine (differentiation medium) supplemented with 0.5 mmol/l 3-isobutyl-1-methyl-xanthine, 1 µmol/l cortisol, 10 µmol/l troglitazone, and 50 µmol/l indomethacin for 7 days. Finally, the cells were allowed to terminally differentiate for another 7 days in differentiation medium without supplements. Medium was changed three times a week. In vitro differentiated adipocytes were washed once with PBS and pretreated overnight with insulin- and FCS-free differentiation medium. Thereafter, cells were incubated for 24 h with or without 300 µg/ml human or bovine fetuin-A, respectively, in insulin- and FCS-free differentiation medium prior to RNA isolation. The procedures were approved by the Ethical Committee of the Tübingen University Medical Department.

### Animal studies

Male C57BL/6 mice were obtained from Charles River Laboratories (Sulzfeld, Germany). For in vivo stimulation, 12-week-old C57BL/6 mice kept on a regular diet obtained an intraperitoneal bolus of bovine fetuin-A (fetuin-I, Pedersen's preparation, purity ∼75%, 0.75 mg/g body weight, ICN, Eschwege, Germany), human fetuin-A (0.5 mg/g body weight), or human serum albumin (0.5 mg/g body weight), respectively. Controls received a comparable amount of diluent (n ≥ 3 each). Serum and perigonadal adipose tissue were removed after 8 h. Tissue samples were stored at 4°C in RNAlater (Ambion, Huntingdon, United Kingdom). All procedures were conducted according to the guidelines of laboratory animal care and were approved by the local governmental commission for animal research. Insulin resistance was estimated by the homeostasis model assessment of insulin resistance (HOMA-IR) and calculated as insulin (µU/ml) · glucose (mM)/22.5 [29].

### Real-time RT-PCR

For quantification of mRNA expression in mouse adipose tissue, RNA was isolated with peqGOLD TriFast according to the manufacturer's instructions (Peqlab, Erlangen, Germany). For quantification of THP1 monocyte and human adipocyte mRNA, cells were washed, harvested, and RNA was isolated with RNeasy silica-gel columns according to the manufacturer's instructions (Qiagen, Hilden, Germany). The total RNA was treated with RNase-free DNase I and transcribed into cDNA using AMV reverse transcriptase and the first strand cDNA kit from Roche Diagnostics (Mannheim, Germany). Quantitative PCR was performed with SYBR Green I dye on a high speed thermal cycler with integrated microvolume fluorometer according to the instructions of the manufacturer (Roche Diagnostics, Mannheim, Germany). Primers were obtained from Invitrogen (Karlsruhe, Germany) and PCR conditions are given in Table 1. Measurements were performed in triplicate. Cellular mRNA contents are given, after correction for 28S-rRNA, in relative arbitrary units (RAU or % of control).

**Table 1:** Conditions for RT-PCR quantification of specific mRNAs and 28S-rRNA.

RNA	Forward primer (5′→3′)	Reverse primer (5′→3′)	T_annealing_ (°C)	No. of cycles	MgCl_2_ (mM)
**Murine**					
*Adipoq* mRNA	GGTGAGACAGGAGATGTTGG	CCTGATACTGGTCGTAGG TG	64	45	3
*Lep* mRNA	ACATTTCACACACGCAGTCG	AGC ATT CAG GGC TAA CAT CC	69	55	5
*Il6* mRNA	GATGCTACCAAACTGGATATAATC	GGTCCTTAGCCACTCCTTCTGTG	65	45	6
*Tnf* mRNA	AAATGGCCTCCCTCTCATCA	AGATAGCAAATCGGCTGACG	63	45	3
*Rbp4* mRNA	GTTTTCTCGTGACCCCAATG	GGAGGTGGGGGAAACTAAAC	68	50	3
28S-rRNA	CCAGTACTTCACTCCTGTCT	TCTAAGAGTGAGCAACGACG	61	45	3
**Human**					
*ADIPOQ* mRNA	GTGATGGCAGAGATGGCAC	AGAGGCTGACCTTCACATCC	65	45	4
*IL6* mRNA	CCAGCTATGAACTCCTTCTC	GCTTGTTCCTCACATCTCTC	63	45	3
*TNF* mRNA	GCGTGGAGCTGAGAGATAAC	GATGTTCGTCCTCCTCACAG	61	45	4
*IL1B* mRNA*	CATGGACAAGCTGAGGAAGA	TTCAACACGCAGGACAGGTA	61	45	4
*PBEF1* mRNA	TCCAGGAAGCCAAAGATGTC	GGCCACTGTGATTGGATACC	61	40	3
*RETN* mRNA	GCCGGATTTGGTTAGCTGAG	CTCATTGATGGCTTCTTCCA	66	40	6
*CCL2* mRNA	GCCTCCAGCATGAAAGTCTC	TGGAATCCTGAACCCACTTC	61	40	3
28S-rRNA	ACG GCG GGA GTA ACT ATG ACT	CTT GGC TGT GGT TTC GCT	63	50	4

* For detection of *IL1B* mRNA, the fluorescent dye-linked probes 5′-AGGTGCTCAGGTCATTCTCCTGG-FL-3′ and 5′-Red640-AGGTCTGTGGGCAGGGAACC-PH-3′ were used instead of SYBR Green.

### Human subjects

A total of 122 individuals were studied. These subjects were at increased risk for type 2 diabetes and participated in an ongoing study [24]. Individuals were recruited from the southern part of Germany and were not related to each other. The participants did not take any medication known to affect glucose tolerance or insulin sensitivity. Informed written consent was obtained from all participants, and the Ethical Committee of the Tübingen University Medical Department had approved the protocol.

### Hyperinsulinemic euglycemic clamp

Insulin sensitivity was determined in 49 human subjects as previously described [24]. In brief, subjects received a primed insulin infusion at a rate of 40 mU·m^−2^·min^−1^ for 2 h. Plasma was drawn every 5 min for determination of plasma glucose, and glucose infusion was adjusted appropriately to maintain the fasting glucose level. An insulin sensitivity index for systemic glucose uptake (ISI; in µmol kg^−1^ min^−1^ pM^−1^) was calculated as the mean infusion rate of glucose (in mol kg^−1^ min^−1^) necessary to maintain euglycemia during the last 40 min of the hyperinsulinemic euglycemic clamp divided by the steady state plasma insulin concentration. The latter was the mean insulin concentration at min 100, 110, and 120 of the clamp (522±19 pM).

### Other analytical procedures

Plasma glucose was determined using a bedside glucose analyzer (glucose oxidase method; Yellow Springs Instruments, Yellow Springs, CO, USA). Plasma insulin was determined by an enzyme immunoassay (Abbott Laboratories, Tokyo, Japan). Serum adiponectin levels in mice and fasting plasma fetuin-A levels in human subjects were measured using commercial enzyme-linked immunosorbent assays (ELISA, BioVendor Laboratory Medicine, Brno, Czech Republic). ELISAs were also used to measure serum concentrations of TNF-α, IL-6, and high sensitivity C-reactive protein (hsCRP, R&D Systems Inc., MN, USA). Fasting plasma adiponectin levels in humans were determined by radioimmunoassay (LINCO Research, St. Charles, MO, USA). Multimeric forms of adiponectin were quantified using an enzyme immunoassay (ALPCO Diagnostics, Salem, NH, USA). Percentage of body fat was assessed by bioelectrical impedance.

### Statistical analyses

Data are given as means±SEM. For 3-group comparisons, data were analyzed by ANOVA followed by Dunnett's test. Statistical comparison between treatment and control group were performed with the Student's t-test. To adjust for the effects of relevant covariates (age, gender, body fat) in the in vivo data, multivariate linear regression analysis was performed using log-transformed data. A p-value <0.05 was considered statistically significant. The statistical software package JMP 4.0 (SAS Institute Inc, Cary, NC, USA) was used.

## Results

### Effect of fetuin-A on cytokine mRNA expression of human THP1 monocytes

**Figure 1:**
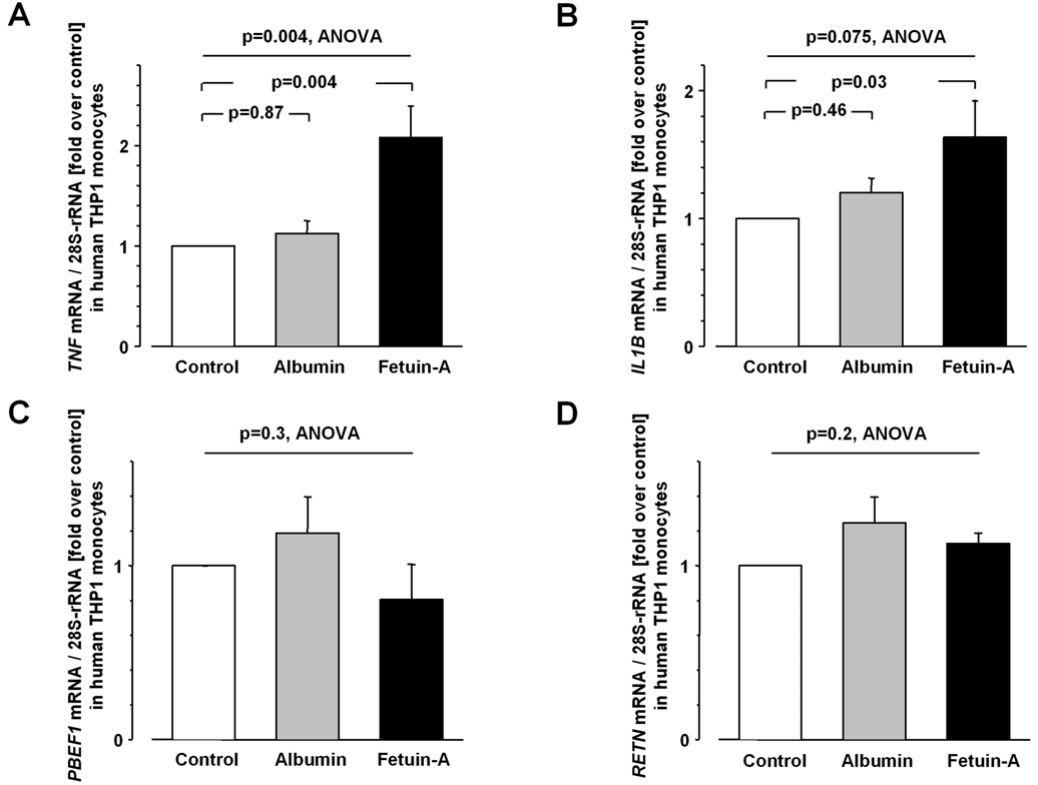
mRNA expression in human THP1 monocytes following fetuin-A treatment. Expression of *TNF* (A), *IL1B* (B), *PBEF1* (C), and *RETN* (D) mRNA in human THP1 monocytes before (control) and after treatment with human albumin or human fetuin-A for 8 h. Cellular mRNA contents were corrected for 28S-rRNA (RAU = relative arbitrary units). Data are given as means±SEM (n = 5). Data were analyzed by ANOVA, followed by Dunnett's test.

Human THP1 monocytes were treated with human fetuin-A and, to evaluate the specificity of fetuin-A's effect and as control for general trophic effects, additionally with human albumin (600 µg/ml, each) for 8 h. Human fetuin-A induced the mRNA expression of *TNF* (ANOVA, F(14,12) = 9.07, p = 0.004, Figure 1A) and *IL1B* (ANOVA, F(14,12) = 4.27, p = 0.075, Figure 1B), but did not modulate mRNA expression of *PBEF1* (encoding visfatin, Figure 1C) or *RETN* (encoding resistin, Figure 1D). Human albumin as a control did not significantly alter the expression of all these genes (Figure 1A–D). The *IL6* mRNA expression in these cells was below the detection limit.

### Effect of fetuin-A on adipose tissue Adipoq mRNA expression and circulating adiponectin in mice

**Figure 2:**
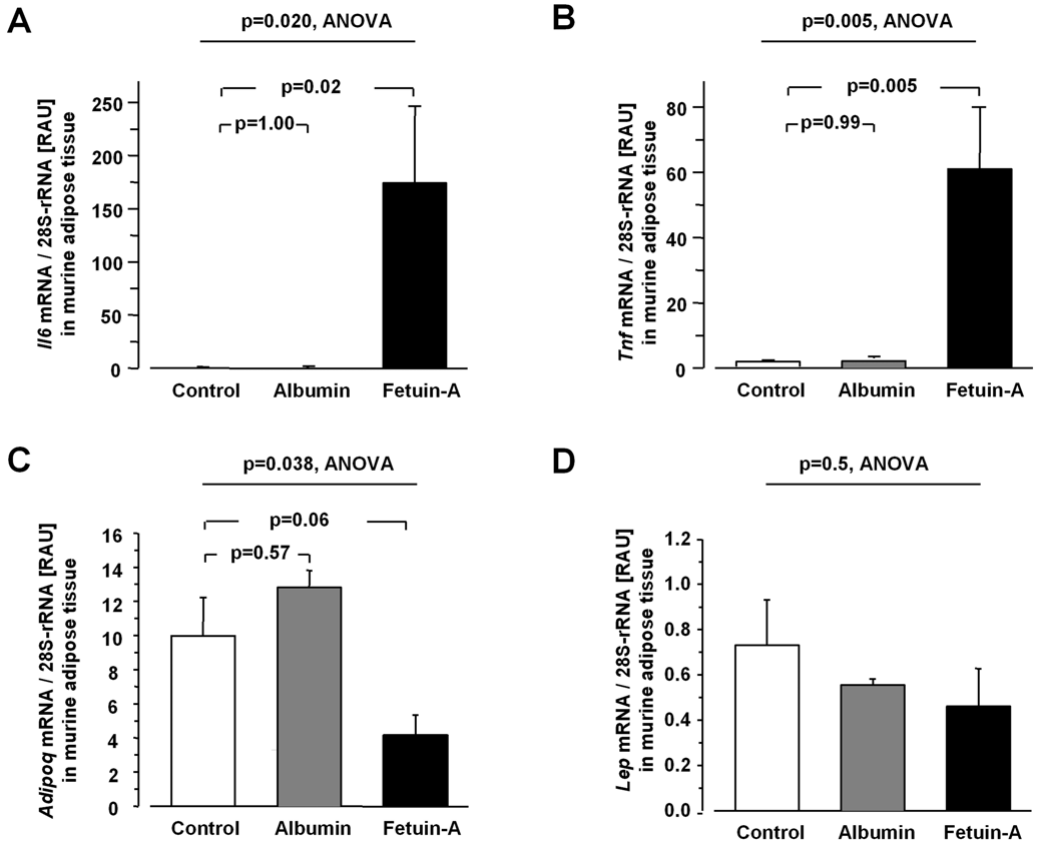
Cytokine and adipokine mRNA expression in adipose tissue of mice. Adipose tissue expression of *Il6* (A), *Tnf* (B), *Adipoq* (C), and *Lep* (D) mRNA in mice after bolus treatment with human fetuin-A (0.5 mg/g body weight), human albumin (0.5 mg/g body weight), or diluent (control) for 8 h. Cellular mRNA contents were corrected for 28S-rRNA (RAU = relative arbitrary units). Data are given as means±SEM. Data were analyzed by ANOVA, followed by Dunnett's test.

Within 8 h, intraperitoneal bolus administration of human fetuin-A (0.5 mg/g body weight) into male C57BL/6 mice resulted in insulin resistance (fetuin-A-treated: 22.46±1.27 vs control: 10.21±1.65, T(4) = 9.06, p = 0.004) as estimated by the HOMA-IR. Furthermore, fetuin-A induced adipose tissue mRNA expression of the inflammatory genes *Il6* and *Tnf* 174- and 27-fold (ANOVA, F(9,7) = 7.12, p = 0.02, and F(9,7) = 12.38, p = 0.005, respectively, Figure 2A,B). This was accompanied by a 58% decrease in adipose tissue *Adipoq* mRNA content as compared to control (ANOVA, F(9,7) = 6.15, p = 0.038, Figure 2C), that showed a strong trend after correction for multiple comparison (p = 0.06). To validate these findings on *Adipoq* mRNA expression, we repeated the experiment and additionally measured serum adiponectin. In the second experiment, treatment of mice with a commercially available bovine fetuin-A (0.75 mg/g body weight) provoked a significant 45% decrease in perigonadal adipose tissue *Adipoq* mRNA content as compared to baseline (T(4) = 4.30, p = 0.006), whereas no effect was seen in diluent-treated control animals (p = 0.48). Concomitantly, serum adiponectin levels significantly decreased in fetuin-A-treated (T(4) = 2.58, p = 0.031), but not in diluent-treated mice (p = 0.41). To assess specificity for adiponectin, we additionally quantified adipose tissue mRNA expression of other adipokine genes such as *Lep* (encoding leptin) and *Rbp4* (encoding retinol-binding protein 4). Fetuin-A did not modulate *Lep* (Figure 2D) or *Rbp4* (data not shown) mRNA expression. Treatment of mice with human albumin (0.5 mg/g body weight) did not significantly alter the expression of all these genes (all p>0.2, Figure 2A–D).

### Effect of fetuin-A on ADIPOQ and cytokine mRNA expression of human in vitro differentiated adipocytes

**Figure 3:**
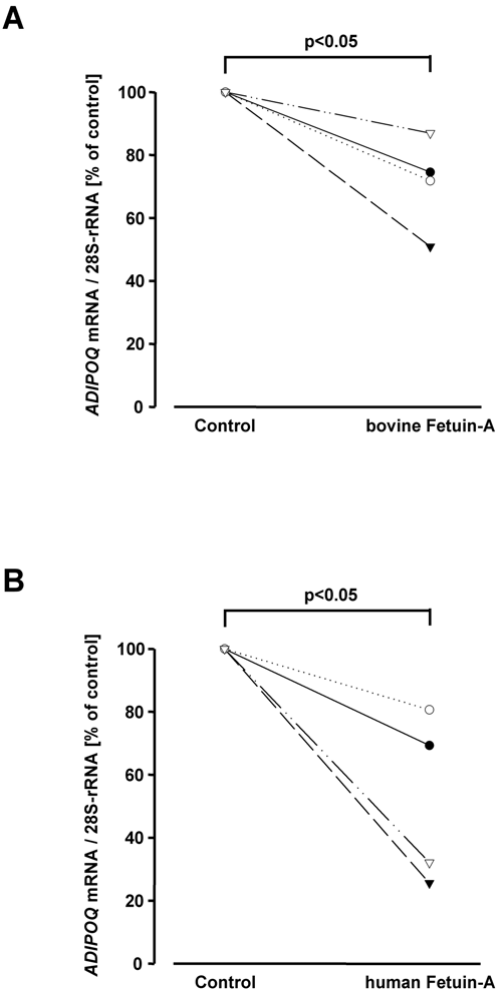
Adiponectin mRNA expression in cultured human adipocytes. *ADIPOQ* mRNA expression of human *in vitro* differentiated adipocytes after treatment with bovine (A) and human (B) fetuin-A for 24 h. Cellular mRNA contents were corrected for 28S-rRNA. Data from adipocyte cultures of four donors are presented. Data were analyzed by Student's t-test.

To test whether suppression of *Adipoq* mRNA expression by fetuin-A is a direct effect on adipocytes and is not restricted to rodents, we treated *in vitro* differentiated human adipocytes from four donors with bovine or human fetuin-A (300 µg/ml) for 24 h. These procedures resulted in 30- and 50-% reductions of *ADIPOQ* mRNA expression, respectively (T(3) = −3.87, p = 0.001 and T(3) = −3.54, p = 0.016, respectively, Figure 3). In analogy to our results in human THP1 monocytes, fetuin-A induced inflammatory markers in adipocytes as well (*TNF*: p = 0.007; *IL6*: p = 0.035; *CCL2*: p = 0.03; *IL1B*: p = 0.068, data not shown). To further investigate whether paracrine or autocrine effects of fetuin-A exist, we determined the fetuin-A mRNA expression in human THP1 monocytes and human adipose tissue and for comparison in human liver. While fetuin-A was expressed in liver tissue from human donors, it was not expressed in THP1 monocytes or adipose tissue (Figure S1). Furthermore, fetuin-A expression was not detected in adipose tissue from obese and insulin resistant Pima Indians (personal communication Dr. Paska Permana) suggesting that these conditions do not induce fetuin-A expression in adipose tissue in humans. Thus, these data strongly support that the endocrine action of fetuin-A mediates the aforementioned effects.

### Relationship of plasma fetuin-A with circulating markers of inflammation and plasma adiponectin

To investigate whether plasma fetuin-A is associated with circulating markers of inflammation and circulating adiponectin in humans *in vivo*, we analyzed cross-sectional data from 122 subjects including 80 subjects with normal glucose tolerance, 40 subjects with impaired glucose tolerance, and two patients with newly diagnosed and untreated type 2 diabetes. The subjects covered a wide range of age, body fat content, waist circumference, and circulating fetuin-A (Table 2). Plasma fetuin-A was not associated with circulating IL-6 and TNF-α (both p ≥ 0.82). This finding was not unexpected since both cytokines act in a paracrine, rather than in a systemic fashion [30]. However, a positive correlation between circulating fetuin-A and hsCRP, a systemic marker of subclinical inflammation, was detected (r = 0.26, p = 0.01, n = 94 with hsCRP above the detection limit of the ELISA). Furthermore, circulating fetuin-A and adiponectin were negatively correlated with each other (r = −0.28, p = 0.02).

**Table 2:** Demographics and metabolic characteristics of the subjects

Variables	Mean±SE	Range
Gender (Males/Females)	50/72	
Age (years)	44±1	19–65
Height (cm)	171±1	149–193
Weight (kg)	85±2	52–166
Waist circumference (cm)	97±1	56–150
Body fat (%)	32±1	10–54
Insulin sensitivity (µM kg^−1^ min^−1^ pM^−1^)	0.064±0.004	0.010–0.200
hsCRP (mg/ml)	0.20±0.03	0.01–1.54
TNF-α (pg/ml)	4.19±0.81	0.06–55.44
Il-6 (pg/ml)	0.93±0.23	0.04–25.93
Total adiponectin (µg/ml)	9.00±0.41	2.92–24.58
Total adiponectin (µg/ml)*	6.43±0.44	2.45–17.47
LMW adiponectin (µg/ml)*	2.36±0.24	0.46–8.83
MMW adiponectin (µg/ml)*	1.49±0.15	0.30–4.86
HMW adiponectin (µg/ml)*	2.58±0.24	0.49–7.81
Fetuin-A (µg/ml)	264±10	111–604

Values are mean (SE);

* available in 49 subjects

Next, we assessed the role of plasma fetuin-A as a determinant of circulating adiponectin and its independence of other known determinants of circulating adiponectin by multivariate linear regression analysis (Table 3). Circulating adiponectin was chosen as the dependent variable, and gender, age, body fat, and waist circumference were used as covariates and added step by step (Table 3, models 1–4). This procedure gradually increased the r^2^ of the model to 0.39 (Table 3, model 4). Inclusion of plasma fetuin-A as an additional covariate further increased the r^2^ to 0.42, and plasma fetuin-A concentration turned out to be an independent determinant of circulating adiponectin (Table 3, model 5).

**Table 3:** Determinants of circulating adiponectin in humans in multivariate linear regression models

Covariates	Estimate±SE	p
**Model 1 (r^2^ = 0.31)**		
Female sex	0.268±0.036	<0.0001
**Model 2 (r^2^ = 0.34)**		
Female sex	0.275±0.036	<0.0001
Age	0.261±0.036	0.03
**Model 3 (r^2^ = 0.38)**		
Female sex	0.335±0.041	<0.0001
Age	0.239±0.116	0.04
Body fat	−1.224±0.441	0.006
**Model 4 (r^2^ = 0.39)**		
Female sex	0.253±0.062	<0.0001
Age	0.268±0.117	0.02
Body fat	−0.376±0.650	0.56
Waist circumference	−0.634±0.359	0.08
**Model 5 (r^2^ = 0.42)**		
Female sex	0.247±0.061	<0.0001
Age	0.187±0.119	0.12
Body fat	−0.293±0.638	0.65
Waist circumference	−0.601±0.352	0.09
Plasma Fetuin-A	−0.234±0.097	0.02

### Relationship between fetuin-A and multimeric forms of adiponectin

**Figure 4:**
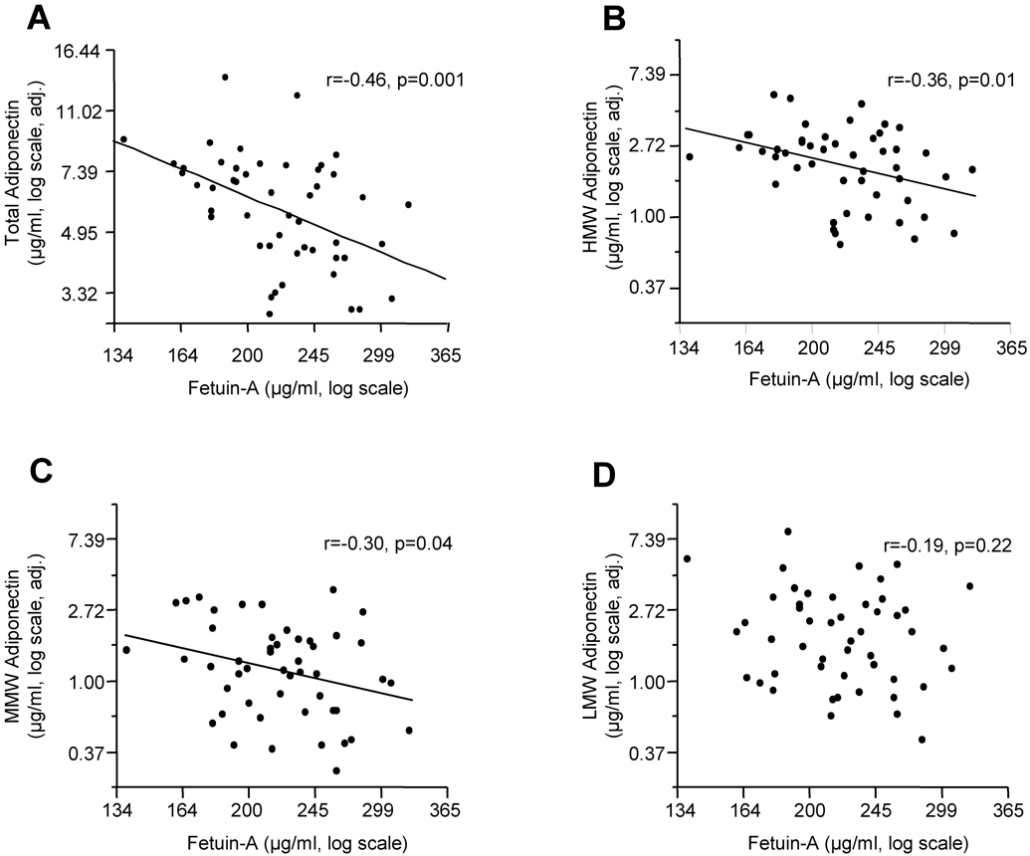
Relationships between circulating fetuin-A and circulating adiponectin in humans. Relationship between plasma levels of fetuin-A with total adiponectin and adiponectin's multimeric forms (A–D) in 49 healthy human subjects after adjustment of log-transformed data for age, sex, and percentage of body fat by multivariate linear regression analysis. The regression coefficients as well as the p-value are indicated (HMW–high molecular weight; LMW–low molecular weight; MMW–middle molecular weight).

Plasma adiponectin circulates in several multimeric forms, i.e. trimeric low molecular weight (LMW), hexameric middle molecular weight (MMW) as well as more complex high molecular weight (HMW) structures [31]. We assessed whether fetuin-A affects the formation of a certain form in a subgroup of 49 subjects who underwent the clamp and had plasma samples stored at −80°C (21 men, 28 women; 23–64 years; 16.0–50.0% body fat; 68–119 cm waist circumference). In this subgroup, total plasma adiponectin was positively correlated with hyperinsulinemic euglycemic clamp-derived insulin sensitivity adjusted for gender, age, and body fat (r = 0.40, p = 0.005). Furthermore, plasma fetuin-A tended to negatively correlate with the adjusted insulin sensitivity (r = −0.25, p = 0.08) reflecting our recent data [24]. Plasma fetuin-A correlated negatively with total adiponectin (Figure 4A) and, in particular, with HMW (Figure 4B) and MMW (Figure 4C) adiponectin, but not with LMW adiponectin (Figure 4D).

## Discussion

Fetuin-A is a major plasma glycoprotein which was discovered in 1944 [32]. For a long time, its biological function remained obscure. Using targeted gene disruption, fetuin-A was recently reported to inhibit ectopic calcification [33], [34]. In keeping with this, fetuin-A deficiency in humans was found to be associated with vascular calcification and mortality in patients on hemodialysis [35]. However, fetuin-A might exert more functions: several studies demonstrated that fetuin-A can act as a natural inhibitor of the insulin receptor tyrosine kinase in liver and skeletal muscle [3]–[7], and fetuin-A knockout mice display improved insulin sensitivity and are resistant to weight gain upon a high-fat diet [8].

Besides the effects of fetuin-A on muscle and liver insulin signalling, there is increasing evidence that fetuin-A is important for insulin action in adipose tissue [10]. Moreover, the variability of plasma adiponectin levels is largely explained by a locus on human chromosome 3q27 harbouring both *ADIPOQ* and, in direct vicinity, *AHSG*. It is of note that not all variability in plasma adiponectin is explained by genetic variation of the *ADIPOQ* gene [23]. This led us to investigate whether fetuin-A regulates adiponectin production and, thus, may explain the recently reported association between fetuin-A and the metabolic syndrome [24], [25]. In addition, the metabolic syndrome and hypoadiponectinemia are strongly associated with low-grade inflammation [27]. Furthermore, high circulating fetuin-A was found to be associated with carotid arterial stiffness [36], a functional property of atherosclerosis that is accompanied by subclinical inflammation. Therefore, we further tested whether fetuin-A treatment affects expression of inflammatory cytokines, a critical step in the generation of low-grade inflammation.

With the present report, we provide novel data that highly purified fetuin-A exerts strong pro-inflammatory effects as it provoked cytokine expression in monocytes *in vitro*. The latter are known to infiltrate hypertrophic adipose tissue and to essentially contribute to adipose tissue inflammation [37], [38]. In addition, we found that, in animals *in vivo,* fetuin-A treatment increased adipose tissue RNA expression of *Il6* and *Tnf* 174- and 27-fold, respectively.

Besides the effects of fetuin-A on the expression of these inflammatory cytokines, administration of fetuin-A into mice repressed *Adipoq* mRNA expression in adipose tissue and decreased circulating adiponectin. *Lep* and *Rbp4* mRNA expression were not affected by fetuin-A, and albumin did not impair *Adipoq* mRNA expression. These findings provide evidence that the effect of fetuin-A on *Adipoq* expression is not due to a general trophic effect [39]. Furthermore, fetuin-A directly affected adipocyte gene expression and these findings were not restricted to rodents: it repressed *ADIPOQ* expression of *in vitro* differentiated human adipocytes.

Based on these findings *in vitro* and in animals, we further investigated whether circulating fetuin-A was related to low-grade inflammation and circulating adiponectin in humans. Indeed, plasma fetuin-A levels correlated positively with hsCRP levels, as reported previously by Ix *et al*. [26].

These findings are somewhat unexpected because CRP is up-regulated in inflammatory states while acute inflammation down-regulates fetuin-A expression in the liver [40]. Whether, the latter effect is transient and/or reflects compensational mechanisms, needs to be determined. Furthermore, plasma fetuin-A levels correlated negatively with plasma adiponectin levels. In addition, besides other determinants of adiponectin levels, plasma fetuin-A levels were identified as a contributor to the variability in circulating adiponectin. Of note, the observed relationships of fetuin-A with adiponectin in humans were not very strong, nevertheless, they remained statistically significant after adjustment for established determinants of plasma adiponectin levels. This finding supports the assumption of Ix *et al.* [26] that the strong relationship between plasma fetuin-A levels and the metabolic syndrome may be a result of fetuin-A-induced suppression of adiponectin production.

In addition, fetuin-A was specifically associated with HMW and MMW forms of adiponectin, but not with the LMW form. HMW adiponectin is reported to be the most active adiponectin form [41] and represents, among all multimeric forms of adiponectin, the major determinant of insulin resistance, an atherogenic lipoprotein profile, and the metabolic syndrome [41]–[43]. How fetuin-A determines the assembly of trimeric adiponectin into hexameric MMW and higher-order HMW structures, a process that is supposed to occur during the entry of adiponectin into the bloodstream [31], is currently unknown and needs further investigation.

So far, no information is available on fetuin-A-specific cell surface receptors and intracellular signaling pathways making it difficult to assess the molecular mechanisms underlying fetuin-A-induced repression of *ADIPOQ* expression in adipocytes as well as fetuin-A-mediated induction of cytokine expression in monocytes and adipocytes. Since the *ADIPOQ* gene is under the control of the transcription factor peroxisome proliferator-activated receptor (PPAR) γ, we also assessed whether PPARγ expression was altered by fetuin-A treatment. Whereas fetuin-A reduced adipose tissue PPARγ expression in mice in vivo, PPARγ expression of adipocytes was not affected (data not shown). The down-regulation of adiponectin and possibly PPARγ expression, appears contradicting to the reported pro-adipogenic effect of fetuin-A [11]. However, this observation may be due to differential effects of fetuin-A on mature adipocytes and pre-adipocytes, respectively.

Since we observed a strong effect of fetuin-A on cytokine expression, and IL-6 and TNF-α are well-described negative regulators of adiponectin expression and production [44]–[49], we suppose that fetuin-A-induced impairment of adiponectin synthesis may be at least partially mediated by effects of fetuin-A on IL-6 and TNF-α production. Whether this is an exclusive mechanism, or whether fetuin-A regulates adiponectin expression and secretion independently of cytokine production, needs to be determined in future studies.

Together, the results presented in this work advance our understanding of the role of fetuin-A in the pathophysiology of insulin resistance, atherosclerosis, and the metabolic syndrome. Hepatic steatosis that is strongly related to insulin resistance and type 2 diabetes [50], associates with enhanced hepatic fetuin-A production in rats [51] and humans [24]. Elevated circulating levels of fetuin-A negatively affect whole-body insulin sensitivity (i) by impairment of insulin signaling in muscle and liver [3]–[7] and (ii) by triggering inflammation in adipose tissue and suppression of adiponectin production. Generation of an atherogenic lipoprotein profile [52], induction of inflammatory cytokines as well as suppression of the atheroprotective hormone adiponectin [53], therefore, represent plausible molecular pathways linking fatty liver and atherosclerosis [54]. In addition, based on the findings from fetuin-A deficient mice that remain lean and insulin sensitive fed a high-fat diet [8], fetuin-A may have long-term effects on energy expenditure and lipid oxidation, independent of the aforementioned mechanisms.

In conclusion, we found that fetuin-A induces low-grade inflammation and represses adiponectin production in animals and in humans. These data provide novel evidence on the role of fatty liver in the pathophysiology of insulin resistance and atherosclerosis.

## Supporting Information

Figure S1 (0.50 MB TIF)
