# Supplementary figures and images for: Fetuin-A Induces Cytokine Expression and Suppresses Adiponectin Production
Source: PLoS One. 2008 Mar 12;3(3):e1765. doi: 10.1371/journal.pone.0001765 (PMC2258416; doi:10.1371/journal.pone.0001765)

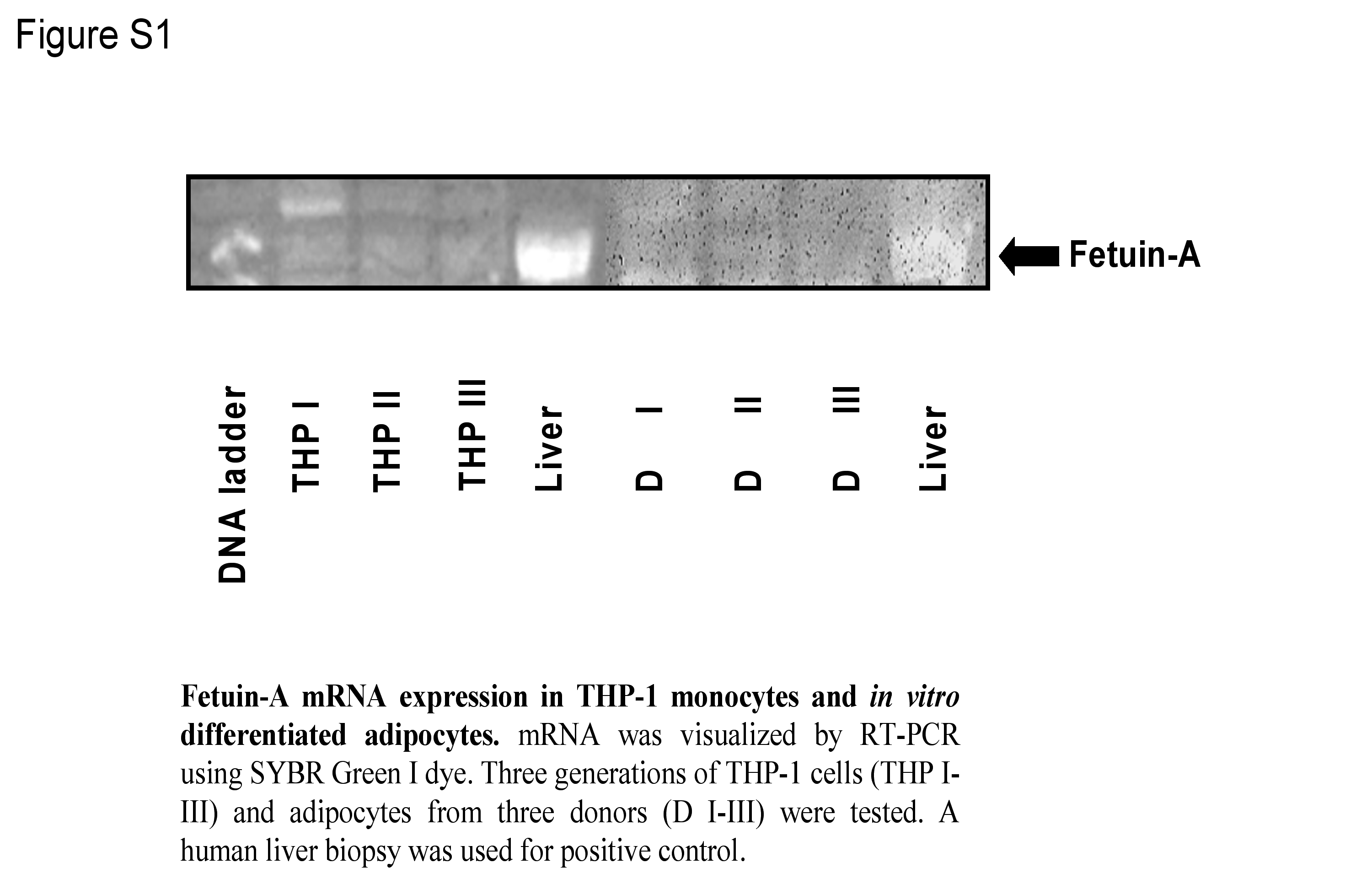

Supplement: Figure S1 — (0.50 MB TIF) [file pone.0001765.s001.tif]
